# Dysregulated neutrophil extracellular trap formation is a novel driver of clonogenicity and therapeutic vulnerability in CMML

**DOI:** 10.64898/2026.07.28.741351

**Published:** 2026-07-29

**Authors:** Saveg Yadav, Callie T. Brown, Mark Cody, William L. Heaton, Claudia de Araujo, Marco Marchetti, Robert A. Campbell, Anthony D. Pomicter, Justin Williams, Christian Con Yost, Shannon E. Elf, Ami B. Patel

## Abstract

Chronic myelomonocytic leukemia (CMML) is an aggressive hematologic malignancy characterized by excess inflammatory signaling and clonal myeloproliferation. The relative contribution of neutrophils (PMNs) to the inflammatory milieu in CMML is poorly understood. In this study we sought to understand whether neutrophil extracellular trap (NET) formation, a key mediator of neutrophilic inflammation, is dysregulated in CMML and can be therapeutically targeted with a novel peptide inhibitor of NETosis called neonatal NET-inhibitory factor (nNIF). Here, we demonstrate that baseline NET formation is aberrantly increased in primary CMML PMNs transcriptionally primed for NETosis, and that soluble factors produced during CMML NET formation promote clonogenicity in CMML CD34 ^+^ hematopoietic cells matched to the same patient. Further, we show that nNIF and clinical agents under investigation in CMML effectively inhibit NETosis, warranting further study of NET inhibitory agents in this rare disease with limited treatment options.

## Full Text Availability

The license terms selected by the author(s) for this preprint version do not permit archiving in PMC. The full text is available from the preprint server.

